# Heartfelt struggles: When ablation brings the squeeze

**DOI:** 10.21542/gcsp.2024.48

**Published:** 2024-11-01

**Authors:** Hussam Al Hennawi, Alexander Shpilman

**Affiliations:** 1 Department of Internal Medicine, Jefferson Abington Hospital, PA, USA; 2 Department of Cardiology, Jefferson Abington Hospital, PA, USA

## Abstract

Pericarditis frequently occurs as a complication following pulmonary vein isolation (PVI) for atrial fibrillation (AF), yet it seldom progresses to constrictive pericarditis (CP). The diagnosis of constrictive pericarditis is often challenging owing to nonspecific signs and symptoms. Nevertheless, a high level of suspicion and prompt diagnosis offer an ultimate cure. We present a case of a 65-year-old patient who developed chronic pericarditis following PVI. This instance underscores the importance of promptly identifying this complication within this particular group of patients.

## Case presentation

A 65-year-old male was evaluated for dyspnea on exertion and fatigue for four months. He had a history of chronic diastolic heart failure, long-standing persistent atrial fibrillation status post six catheter ablations, most recently 4 months before presentation, followed by cardiac resynchronization therapy and coronary artery disease status post percutaneous intervention, obstructive sleep apnea, hypertension, hyperlipidemia, and diabetes mellitus. Examination revealed jugular venous distention and trace bilateral lower extremity edema.

**Figure 1.**
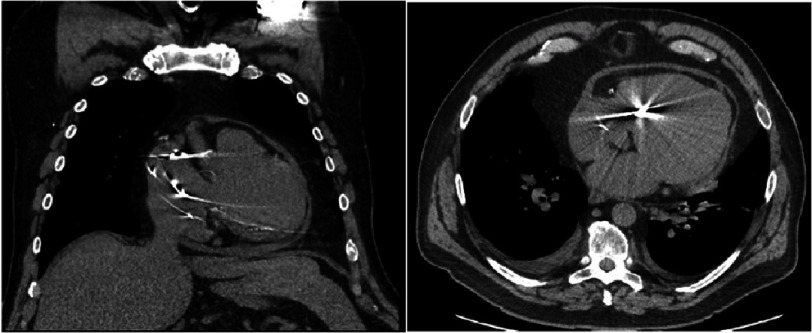
Computerized chest tomography showed a small pericardial effusion with a noncalcified thickened pericardium.

He was taking amlodipine 10 mg, atorvastatin 40 mg, bumetanide 1 mg twice daily, clopidogrel 75 mg, dapagliflozin 10 mg, metoprolol succinate 100 mg twice daily, and warfarin. Transthoracic echocardiogram showed markedly dilated LV with normal wall thickness and mild-moderately decreased systolic function (compared to echocardiogram prior to PVI) with regional variation and dyssynchrony of contractility, excessive respiratory phasic septal motion, and inferior vena cava appeared dilated with reduced inspiratory decrease. Computerized tomography showed a small pericardial effusion with a noncalcified thickened pericardium (Figure 1).

**Figure 2.**
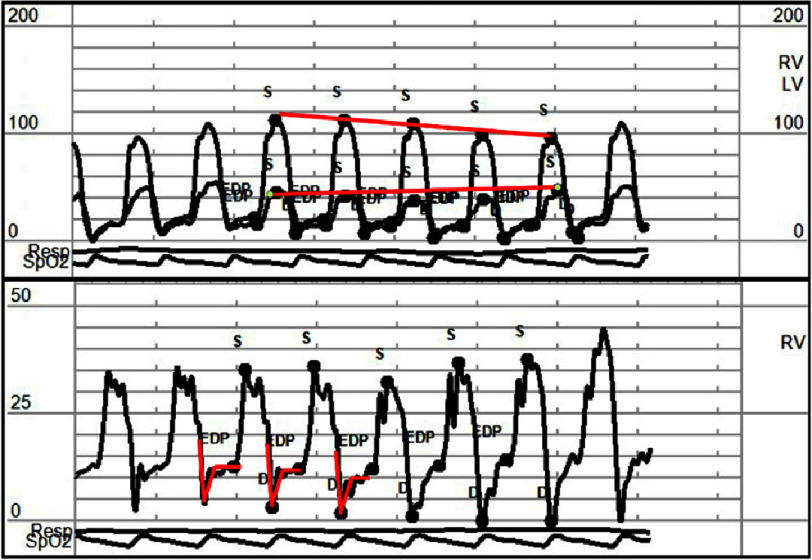
Right and left heart catheterization filling pressure curves show equalization of right and left ventricles, diastolic filling pressures, and a “dip and plateau” sign in the right ventricle pressure tracing (red square root sign). Solid lines show left and right ventricular pressure respirophasic discordance.

Right and left heart catheterization showed stable coronary artery disease and a pronounced x and y descent on right atrial tracing, equalization of diastolic pressures with dip and plateau sign (square root sign), and LV/RV pressure respirophasic discordance suggestive of constrictive pericarditis (Figure 2).

C-reactive protein was 3.97 mg/L (reference range 0.00–3 mg/L). Antinuclear antibody and Quantifieron-TB were negative. A diagnosis of chronic constrictive pericarditis was made. He was started on colchicine, but his symptoms did not improve significantly. Owing to persistent symptoms, he underwent pericardial stripping with a resolution of his symptoms.

## Discussion

CP is a rare contributor to heart failure, marked by the heart being surrounded by a thickened, inflexible, and fibrous pericardium. This unyielding enclosure hampers the filling of the ventricles during relaxation (diastole) and leads to interdependence between the ventricles, culminating in reduced cardiac output^1^. Diagnosing CP can be challenging due to vague symptoms, contradictory test outcomes, and its gradual onset^2^.

Although recent literature emphasizes noninvasive multimodality imaging as essential for diagnosing CP, our experience suggests that it often falls short in atypical cases^3^. Therefore, a comprehensive evaluation should include invasive hemodynamic profiling *via* thorough left and right heart catheterization, which remains the gold standard. In the case under review, pressure waveform analysis supported a diagnosis of constrictive pathology, evidenced by a clear square root sign, complete diastolic pressure equalization, and ventricular interdependence with the characteristic respiratory discordance between left ventricular and right ventricular pressure waves^4^.

Given our patient complex history of uncontrolled atrial fibrillation for which he underwent multiple PVI, similar other case reports reported no major difference in terms of CP complication following atrial fibrillation ablation modalities, including radiofrequency ablation *versus* cryoablation^5–8^.

Our case highlights and emphasizes the course of a patient with suspected CP following PVI and encourages early consideration of pericarditis following atrial fibrillation ablation techniques.

## What have we learned?

• Constrictive pericarditis is a rare complication following atrial fibrillation ablation.
• Maintaining a low threshold for diagnosis is essential to ensure timely testing and early intervention.

## Competing Interests

The authors have no competing interests to declare.
